# Nondegenerate Polycrystalline Hydrogen-Doped Indium Oxide (InO*_x_*:H) Thin Films Formed by Low-Temperature Solid-Phase Crystallization for Thin Film Transistors

**DOI:** 10.3390/ma15010187

**Published:** 2021-12-27

**Authors:** Taiki Kataoka, Yusaku Magari, Hisao Makino, Mamoru Furuta

**Affiliations:** 1 Materials Science and Engineering Course, Kochi University of Technology, Kami 782-8502, Kochi, Japan; myyetter97@gmail.com; 2 Graduate School of Natural Science and Technology, Shimane University, Matsue 690-8504, Shimane, Japan; magari.yusaku@riko.shimane-u.ac.jp; 3 Center for Nanotechnology, Research Institute, Kochi University of Technology, Kami 782-8502, Kochi, Japan; makino.hisao@kochi-tech.ac.jp; 4 Electronic and Photonic Systems Engineering Course, Kochi University of Technology, Kami 782-8502, Kochi, Japan

**Keywords:** indium oxide, thin film transistor, solid-phase crystallization, metal–insulator transition

## Abstract

We successfully demonstrated a transition from a metallic InO*_x_* film into a nondegenerate semiconductor InO*_x_*:H film. A hydrogen-doped amorphous InO*_x_*:H (a-InO*_x_*:H) film, which was deposited by sputtering in Ar, O_2_, and H_2_ gases, could be converted into a polycrystalline InO*_x_*:H (poly-InO*_x_*:H) film by low-temperature (250 °C) solid-phase crystallization (SPC). Hall mobility increased from 49.9 cm^2^V^−1^s^−1^ for an a-InO*_x_*:H film to 77.2 cm^2^V^−1^s^−1^ for a poly-InO*_x_*:H film. Furthermore, the carrier density of a poly-InO*_x_*:H film could be reduced by SPC in air to as low as 2.4 × 10^17^ cm^−3^, which was below the metal–insulator transition (MIT) threshold. The thin film transistor (TFT) with a metallic poly-InO*_x_* channel did not show any switching properties. In contrast, that with a 50 nm thick nondegenerate poly-InO*_x_*:H channel could be fully depleted by a gate electric field. For the InO*_x_*:H TFTs with a channel carrier density close to the MIT point, maximum and average field effect mobility (μ_FE_) values of 125.7 and 84.7 cm^2^V^−1^s^−1^ were obtained, respectively. We believe that a nondegenerate poly-InO*_x_*:H film has great potential for boosting the μ_FE_ of oxide TFTs.

## 1. Introduction

Transparent metal oxide semiconductors (OSs) have been extensively investigated for use as the active channel layer of thin film transistors (TFTs) for next-generation flat-panel displays [1,2,3], nonvolatile memories [4,5], image sensors [6,7], and pH sensors [8,9], to name a few. Among OSs, the amorphous In–Ga–Zn–O (IGZO) [10] has attracted particular attention for TFT applications owing to its high field effect mobility (μ_FE_) of more than 10 cm^2^V^−1^s^−1^, steep subthreshold swing (*S.S.*), extremely low off-state current, large-area uniformity, and good bias stress stability [11]. Although the μ_FE_ of an IGZO TFT is approximately one order of magnitude higher than that of an amorphous Si TFT, further improvement of the μ_FE_ of OS TFTs is required to expand their range of applications as an alternative to polycrystalline Si TFT.

Single-crystalline In_2_O_3_ has a Hall mobility as high as 160 cm^2^V^−1^s^−1^ [12], which makes amorphous (a-) or polycrystalline (poly-) InO*_x_* a potential material for enhancing the μ_FE_ of OS TFTs. However, it is known that undoped InO*_x_* thin films exhibit a high background electron density, which is attributed to the presence of native defects, such as oxygen vacancies, making them unsuitable for a channel material of OS TFTs. To reduce the background carrier density, a small amount of a carrier suppressor, having a high bond dissociation energy with oxygen, such as W, Si, or Ti, is doped into an a-InO*_x_* thin film [13,14]. μ_FE_ values of 32, 30, and 17 cm^2^V^−1^s^−1^ were reported for the TFTs with a-InO*_x_*:Ti, W, and Si channels, respectively [13]. An atomic layer deposition (ALD) method is also used to form a- or poly-InO*_x_* channels for TFTs [15,16,17,18]. The TFT with a 5 nm thick ALD-deposited carbon-doped a-InO*_x_*channel with μ_FE_ of 20.4 cm^2^V^−1^s^−1^ has been demonstrated [18]. Higher μ_FE_ values of 39.2 and 41.8 cm^2^V^−1^s^−1^ were reported for the TFTs with ultrathin (5 nm) poly-InO*_x_* channels formed by plasma- or ozone-assisted ALD followed by postdeposition annealing (PDA). [15,16], A poly-InO*_x_* film is also known to be deposited by sputtering even without substrate heating. A fully depleted poly-InO*_x_* TFT with μ_FE_ of 15.3 cm^2^V^−1^s^−1^ was obtained by decreasing the channel thickness to 8 nm. [19] Most a- and poly-InO*_x_* TFTs have been demonstrated with an ultrathin (<10 nm) channel layer [13,14,15,16,18,19], in order to fully deplete degenerate InO*_x_* channels. However, an ultrathin poly-InO*_x_* channel layer limits the μ_FE_ of the TFTs, since the grain size of the film is small. Poly-InO*_x_* films have also been investigated for use as the transparent conductive oxide (TCO) in solar cells. Koida et al. [20,21,22,23,24,25] reported a hydrogen-doped poly-InO*_x_* (InO*_x_*:H) film with high electron mobility and high near-infrared (NIR) transparency prepared by solid-phase crystallization (SPC) [20]. To incorporate H-donors into InO*_x_*, H_2_O vapor or H_2_ gas is introduced during sputtering deposition. During the PDA of an InO*_x_*:H film, phase transition from amorphous to polycrystalline (SPC) occurred at ~175 °C. The SPC poly-InO*_x_*:H films showed a Hall mobility as high as 100–130 cm^2^V^−1^s^−1^; however, its carrier density (>1 × 10^20^ cm^−3^) was too high to apply it as a channel layer of the TFT [20]. Thus, for the TFT application, the carrier density should be reduced to obtain a nondegenerate semiconductor InO*_x_*:H film. We previously reported the electrical properties of the H-doped a-IGZO (IGZO:H) prepared by sputtering in Ar, O_2_, and H_2_ gases [26,27,28,29]. Although the as-deposited IGZO:H was degenerate semiconductor with the carrier density of over 1 × 10^20^ cm^−3^, carrier density significantly decreased more than two orders of magnitude after PDA at 150 °C in air.

In this work, nondegenerate poly-InO*_x_*:H thin films were successfully prepared by SPC. A degenerate a-InO*_x_*:H thin film was deposited by sputtering in Ar, O_2_, and H_2_ gases, and an amorphous to polycrystalline phase transition of the film was achieved after PDA at more than 175 °C. By PDA at 250 °C in air, a nondegenerate poly-InO*_x_*:H film could be obtained with a carrier density as low as 2.4 × 10^17^ cm^−3^, which is approximately three orders of magnitude lower than that of the initial a-InO*_x_*:H film. The TFTs with a 50 nm thick nondegenerate InO*_x_*:H channel could be fully depleted by a gate electric field. A maximum μ_FE_ of 125.7 cm^2^V^−1^s^−1^ was exhibited by the TFT with the poly-InO*_x_*:H channel. The use of a nondegenerate poly-InO*_x_*:H film is a promising approach to boost the μ_FE_ of OS TFTs.

## 2. Experiments

Indium oxide (InO*_x_*) and hydrogenated InO*_x_* (InO*_x_*:H) films with a thickness of 50 nm were deposited on a glass substrate by radio frequency (RF) magnetron sputtering, without intentional substrate heating, from a ceramic In_2_O_3_ target. Mixed gases of Ar/O_2_ and Ar/O_2_/H_2_ were used for depositing InO*_x_* and InO*_x_*:H films, respectively. The O_2_ gas flow ratio, which was defined as R[O_2_] = O_2_/(Ar + O_2_+ H_2_), was set at 4% for both films, while the H_2_ gas flow ratio, which was defined as R[H_2_] = H_2_/(Ar + O_2_ + H_2_), was set at 1, 5, and 9% for InO*_x_*:H films. All the films were deposited at 0.4 Pa and then annealed at temperatures ranging from 100 to 400 °C in either N_2_ or ambient air. The carrier concentration (*N*_e_) and Hall mobility (μ_H_) of the films were measured by Hall effect measurements using van der Pauw geometry. An absorption coefficient (α) of the films was evaluated from transmittance and reflectance measurements. The crystallinity and grain size of the films were evaluated by X-ray diffraction (XRD) analysis and scanning electron microscopy (SEM), respectively. The amounts of hydrogen and hydroxyl groups in the films were measured by thermal desorption spectroscopy (TDS). The chemical composition (In/O ratio) and hydrogen content in the films were measured by Rutherford backscattering spectrometry (RBS) and elastic recoil detection analysis (ERDA), respectively. The chemical bonding states of the films were evaluated by a custom-made, hard X-ray photoelectron spectroscopy (HXPS) system with a CrKα X-ray source of 5415 eV and a wide acceptance angle electron analyzer.

## 3. Results and Discussion

### 3.1. Optical, Electrical, and Structural Properties of As-Deposited InO_x_ and InO_x_:H Films

**Figure 1:**
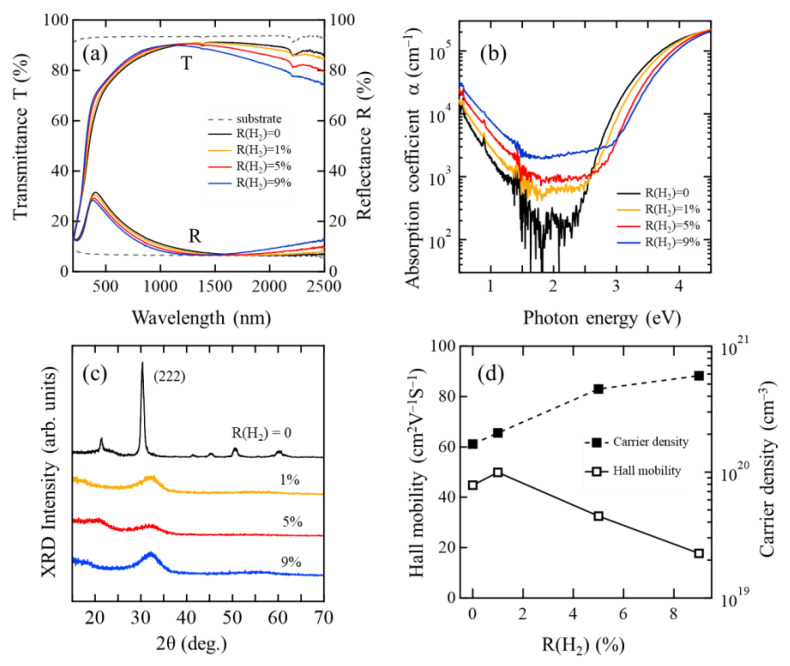
(**a**) Transmittance and reflectance, (**b**) absorption coefficient, (**c**) XRD spectra, and (**d**) Hall mobility and carrier density of as-deposited poly-InO*_x_* [R(H_2_) = 0] and a-InO*_x_*:H [R(H_2_) = 1, 5, 9%] films.

Figure 1a shows the transmittance and reflectance of the as-deposited InO*_x_* and InO*_x_*:H films. The absorption edge of the InO*_x_*:H films shifted to a shorter wavelength as R(H_2_) increased. On the other hand, the transmittance of the InO*_x_*:H films decreased in the NIR region with increasing R(H_2_). Figure 1b shows the absorption coefficient (α) of the as-deposited InO*_x_* and InO*_x_*:H films as a function of photon energy. The subgap α of the InO*_x_*:H films increased with R(H_2_). Figure 1c shows XRD spectra of the as-deposited InO*_x_* and InO*_x_*:H films. An InO*_x_* film showed a clear polycrystalline nature with a (222) preferred orientation, whereas all the InO*_x_*:H films with R(H_2_) from 1 to 9% exhibited an amorphous nature. These results indicate that added H_2_ suppressed crystallization in the vapor phase during the deposition and formed the a-InO*_x_*:H film. Figure 1d shows the μ_H_ and *N*_e_ of the as-deposited poly-InO*_x_* and a-InO*_x_*:H films as a function of R(H_2_), during the deposition. The poly-InO*_x_* film showed μ_H_ of 44.8 cm^2^V^−1^s^−1^. On the other hand, the μ_H_ of a-InO*_x_*:H film increased to 49.9 cm^2^V^−1^s^−1^ at R(H_2_) of 1%; however, it decreased to 17.7 cm^2^V^−1^s^−1^ as R(H_2_) further increased to 9%. Since hydrogen acts as a shallow donor in the films, *N*_e_ monotonically increased from 1.7 × 10^20^ cm^−3^ for poly-InO*_x_* to 5.8 × 10^20^ cm^−3^ for a-InO*_x_*:H, upon R(H_2_) increasing to 9%. Thus, the transmittance of the a-InO*_x_*:H films decreased in the NIR region owing to a free carrier absorption. The increases in subgap α and *N*_e_, and the decrease in the μ_H_ of the as-deposited a-InO*_x_*:H films suggest that H_2_ added during film deposition generates defects such as oxygen vacancies (V_O_), owing to sputtering in a reducing atmosphere.

### 3.2. Changes in Film Properties through PDA

**Figure 2:**
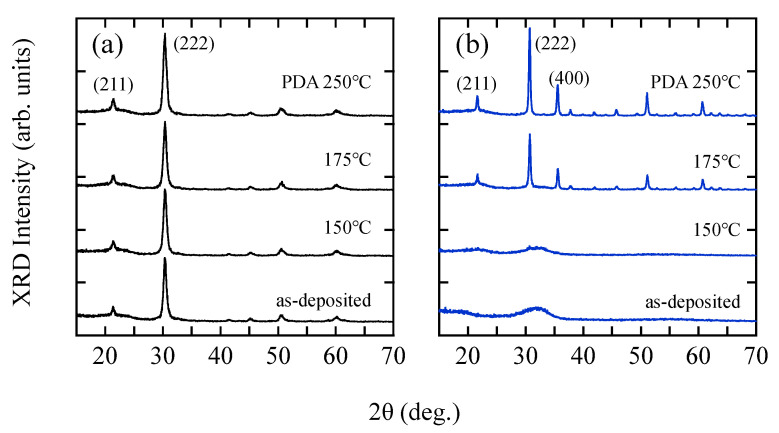
Changes in XRD spectra of (**a**) InO*_x_* and (**b**) InO*_x_*:H [R(H_2_) = 5%] films with PDA at different temperatures.

Figure 2 shows XRD spectra of (a) InO*_x_* and (b) InO*_x_*:H [R(H_2_) of 5%] films before (as-deposited) and after PDA at 150, 175, and 250 °C for 1 h. An InO*_x_* film showed a clear polycrystalline nature with a (222) preferred orientation even before annealing (as-deposited), whereas the InO*_x_*:H film remained amorphous even after PDA at 150 °C. The a-InO*_x_*:H film could be converted to a poly-InO*_x_*:H film when the PDA temperature was raised to more than 175 °C. In this paper, we define the amorphous to polycrystalline phase transition upon PDA as SPC. In addition, the full width at half maximum (FWHM) of the poly-InO*_x_*:H film was smaller than that of the poly-InO*_x_* film after PDA at 250 °C (data not shown here), indicating a higher crystallinity of the poly-InO*_x_*:H film than of the poly-InO*_x_* film. The In/O ratio of the films after PDA at 250 °C were 0.51 for poly-InO*_x_* film and 0.55 poly-InO*_x_*:H film, indicating that both films contain higher O content than the stoichiometric film (In/O ratio of 0.67). The atomic ratio of hydrogen in the poly-InO*_x_*:H film was estimated to be 8.6% after the SPC process at 250 °C.

**Figure 3:**
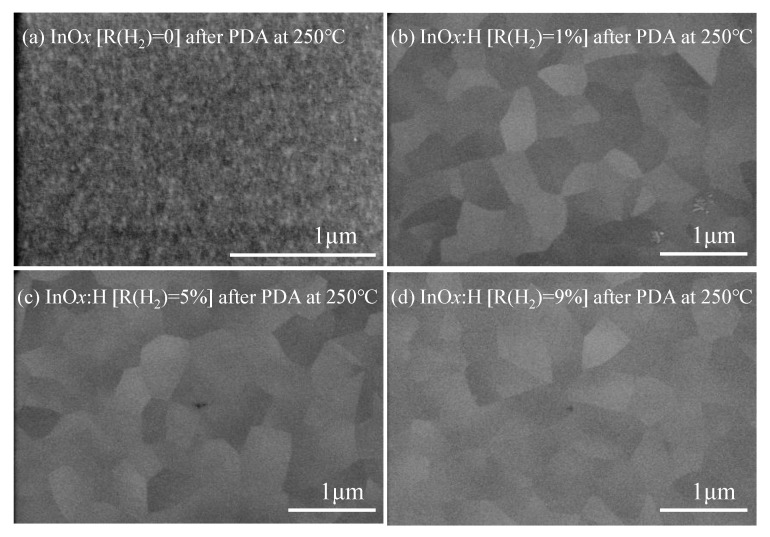
SEM surface views of (**a**) poly-InO*_x_* [R(H_2_) = 0] and poly-InO*_x_*:H [R(H_2_) = (**b**) 1, (**c**) 5, and (**d**) 9%] films after PDA at 250 °C.

Figure 3 shows SEM views of the poly-InO*_x_* and poly-InO*_x_*:H film surfaces after PDA at 250 °C. The poly-InO*_x_* film showed very fine grains. In contrast, the grain size of the poly-InO*_x_*:H film significantly increased to ~400 nm, owing to SPC from the a-InO*_x_*:H film. Moreover, the grain size of the poly-InO*_x_*:H film did not depend on R(H_2_).

**Figure 4:**
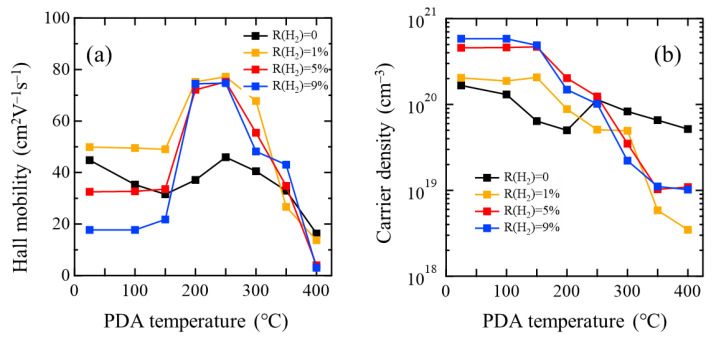
(**a**) Hall mobility and (**b**) carrier density of InO*_x_* [R(H_2_) = 0] and InO*_x_*:H [R(H_2_) = 1, 5, and 9%] films as a function of PDA (in N_2_) temperature.

Figure 4 shows (a) μ_H_ and (b) *N*_e_ values of the InO*_x_* and InO*_x_*:H films as a function of the temperature of the PDA, which was applied in N_2_ for 1 h. μ_H_ of the poly-InO*_x_* film (without H_2_) after PDA at 250 °C was 46.0 cm^2^V^−1^s^−1^, which was almost unchanged from that of the as-deposited InO*_x_* film. In contrast, the μ_H_ of all the InO*_x_*:H films sharply increased after PDA at 200 °C, which is in good agreement with the phase transition temperature from amorphous to polycrystalline, as shown in Figure 2b. Furthermore, all the poly-InO*_x_*:H films with R(H_2_) values of 1, 5, and 9% exhibited almost the same μ_H_ because their grain sizes were almost the same, as shown in Figure 3. Thus, the enlargement of the grain size and the improvement of intragrain crystallinity due to SPC are the main causes of the improved μ_H_ of the poly-InO*_x_*:H films after PDA at 200 °C. Maximum μ_H_ values of 74.8–77.2 cm^2^V^−1^s^−1^ were obtained from the poly-InO*_x_*:H films after PDA at 250 °C. On the other hand, the *N*_e_ values of as-deposited poly-InO*_x_* and a-InO*_x_*:H [R(H_2_) of 9%] films were 1.7 × 10^20^ cm^−3^ and 5.8 × 10^20^ cm^−3^, respectively. Although the *N*_e_ of the films slightly decreased after PDA at 250 °C, all the poly-InO*_x_*:H films were still degenerate with *N*_e_ values of 5.1 × 10^19^–1.2 × 10^20^ cm^−3^.

**Figure 5:**
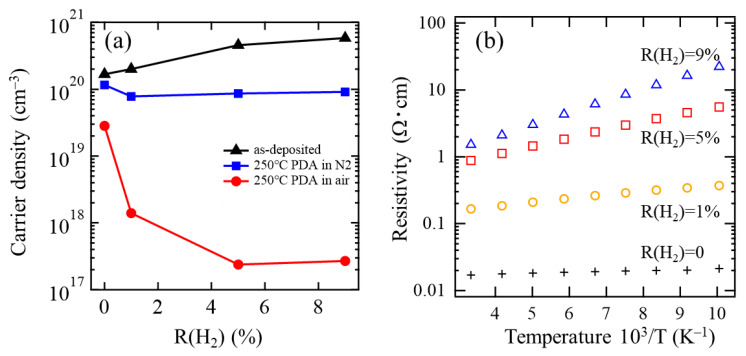
(**a**) Carrier density of InO*_x_* [R(H_2_) = 0] and InO*_x_*:H [R(H_2_) = 1, 5, 9%] films before (as-deposited) and after 250 °C PDA (in both N_2_ and air) as a function of R(H_2_). (**b**) Temperature dependence of resistivity of InO*_x_* [R(H_2_) = 0] and InO*_x_*:H [R(H_2_) = 1, 5, 9%] films after PDA at 250 °C in air.

Since *N*_e_ values of N_2_-annealed poly-InO*_x_* and poly-InO*_x_*:H films are too high for these films to be used as a channel layer of the TFT, the annealing ambient was changed from N_2_ to air to reduce the *N*_e_ of the films. Figure 5a shows the *N*_e_ values of the as-deposited and 250 °C annealed poly-InO*_x_* and InO*_x_*:H films as a function of R(H_2_) during film deposition. As mentioned previously, the *N*_e_ of as-deposited films increased with R(H_2_). After PDA at 250 °C in N_2_ (same as shown in Figure 4b), all the films showed mostly the same *N*_e_ of approximately 10^20^ cm^−3^, regardless of R(H_2_). On the other hand, after PDA at 250 °C in air, the *N*_e_ of the InO*_x_* film decreased to 2.8 × 10^19^ cm^−3^, while those of the poly-InO*_x_*:H films further decreased from 1.4 × 10^18^ cm^−3^ to 2.7 × 10^17^ cm^−3^ as R(H_2_) increased from 1 to 9%. The *N*_e_ of the poly-InO*_x_*:H films annealed in air decreased by more than two orders of magnitude from that of the films annealed in N_2_. This result indicates that both the H_2_ added during the film deposition and the oxygen in the annealing ambient play an important role in reducing the *N*_e_ of the films. The Hall measurement results presented in Figure 5a are also summarized in Table 1.

The critical carrier density of the metal–insulator transition (MIT) is given by the Mott criterion *n*_c_ = (0.26/*a*_H_)^3^, where *a*_H_ denotes the effective Bohr radius of a hydrogenic donor [29]. For In_2_O_3_ with a relative dielectric constant (ε) of 9, *n*_c_ is calculated to be 1.6 × 10^18^–7.2 × 10^18^ cm^−3^ when the electron effective mass (m*/*m*_0_) is assumed to be 0.1–0.3 [30,31,32]. Thus, a nondegenerate semiconductor film would be obtained from the InO*_x_*:H films.

Figure 5b shows the inverse temperature (1000/T) dependence of the resistivity (ρ) of the films after PDA at 250 °C in air. For the poly-InO*_x_* film [R(H_2_) = 0], ρ did not change in the temperature range from 100 to 300 K, indicating that the film exhibited degenerate metallic conduction. On the other hand, the ρ of the poly-InO*_x_*:H with R(H_2_) of 1% increased from 1.7 × 10^−2^ to 5.1 × 10^−2^ Ω·cm when the measurement temperature was decreased from 300 to 100 K. The positive correlation of ρ and inverse temperature further increased for the films with R(H_2_) values of 5 and 9%. This result clearly indicated that nondegenerate semiconductor poly-InO*_x_*:H films could be obtained by SPC in air.

### 3.3. TDS and HXPS Analysis of InO_x_ and InO_x_:H Films

**Figure 6:**
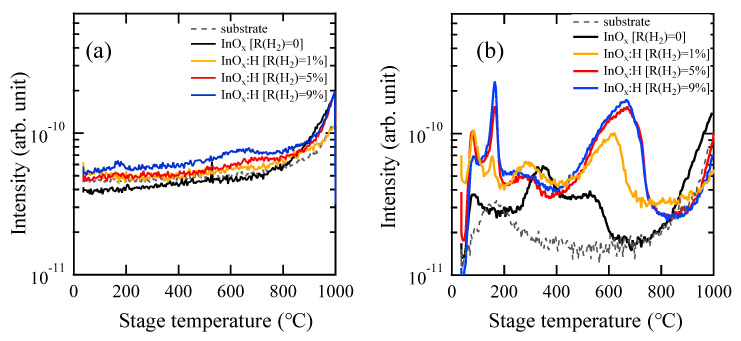
TDA spectra of (**a**) *m*/*z* = 2 (H_2_) and (**b**) *m*/*z* = 18 (H_2_O) obtained from as-deposited InO*_x_* [R(H_2_) = 0] and InO*_x_*:H [R(H_2_) = 1, 5, 9%] films.

An introduced H_2_ in the InO*_x_*:H films was evaluated by a thermal desorption spectroscopy (TDS). Figure 6 presents TDS spectra of mass-to-charge ratios (*m*/*z*) of (a) 2 (H_2_) and (b) 18 (H_2_O) obtained from the as-deposited poly-InO*_x_* and a-InO*_x_*:H films. No clear desorption peak of H_2_ was observed from either the InO*_x_* or InO*_x_*:H film, whereas several H_2_O desorption peaks were observed from both types of film, indicating that the introduced H_2_ exists within the hydroxyl (–OH) bonds in the InO*_x_*:H films. The first H_2_O desorption peak due to adsorbed H_2_O molecules at the sample surface was observed at around 80 °C. The second H_2_O desorption peak at around 165 °C was observed only from the InO*_x_*:H films and is attributed to the desorption of hydroxyl (–OH) bonds in the films during an amorphous to polycrystalline phase transition. We also confirmed a small amount of argon desorption from the InO*_x_*:H films at the same temperature as the second peak (data not shown) [21]. The H_2_O desorption observed at a temperature higher than 250 °C can be attributed to the remaining −OH bonds in the films after PDA at 250 °C. TDS results indicated that the number of remained −OH bonds in the InO*_x_*:H film increased as R(H_2_) increased from 1 to 5%, and it saturated at R(H_2_) values of 5 and 9%.

**Figure 7:**
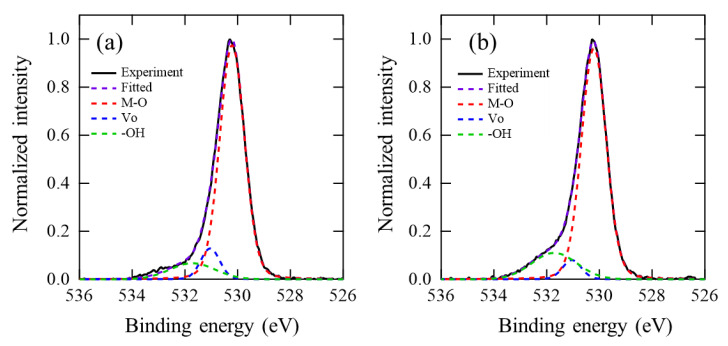
O 1s HXPS spectra obtained from (**a**) poly-InO*_x_* and (**b**) poly-InO*_x_*:H (R[H_2_] = 5%) films after PDA in air at 250 °C.

Figure 7 shows O 1s HXPS spectra obtained from (a) poly-InO*_x_* and (b) poly-InO*_x_*:H (R[H_2_] = 5%) films after PDA at 250 °C, respectively. The O 1s spectra were well fitted by three Gaussian–Lorentz curves at 530.2, 531.0, and 531.7 eV, attributed to the metal–oxygen bonds (M–O), oxygen vacancies (V_O_), and oxygen in the hydroxides (−OH), respectively [29]. The relative area ratio of −OH increased from 9.7% for poly-InO*_x_* film to 15.8% for poly-InO*_x_*:H film, whereas that of V_O_ reduced from 8.2% for poly-InO*_x_* film to 5.0% for poly-InO*_x_*:H film. The HXPS result clearly revealed that hydrogen remained in poly-InO*_x_*:H film as −OH bonds and reduced V_O_ in the film.

By comparing the TDS and HXPS results of the remaining −OH bonds and the *N*_e_ of the InO*_x_*:H films, as shown in Figure 5a, we conclude that the remaining −OH bonds in the film after PDA play an important role in the passivation of oxygen vacancies, which results in the decreasing *N*_e_ of the InO*_x_*:H film.

### 3.4. TFT Application of Polycrystalline InO_x_ and InO_x_:H Films

Bottom-gate poly-InO*_x_* and poly-InO*_x_*:H TFTs were fabricated on a heavily doped n^+^-Si substrate with a 100 nm thick thermally grown SiO_2_ (th-SiO_2_) layer. The Si substrate and th-SiO_2_ were used as the gate electrode and gate insulator (GI) for the TFTs, respectively. The 50 nm thick poly-InO*_x_* and a-InO*_x_*:H films were deposited by sputtering on a GI as a channel layer using a metal mask. R(O_2_) was set at 4% for both films, while R(H_2_) was set at 1, 5, and 9% for the a-InO*_x_*:H film. After the deposition, PDA was applied in both films at 250 °C for 1 h in air. Source and drain electrodes of Au were formed by vacuum evaporation using a metal mask. Finally, TFTs were post-annealed at 200 °C for 1 h in air. The channel length and width of the TFTs were 1000 and 350 μm, respectively.

**Figure 8:**
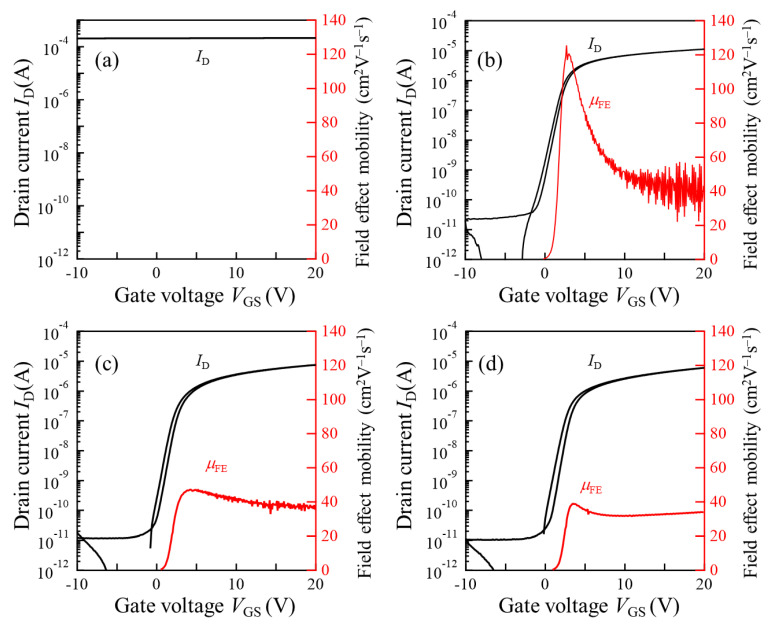
Transfer characteristics and field effect mobility of TFTs with (**a**) InO*_x_* [R(H_2_) = 0] and InO*_x_*:H [R(H_2_) = (**b**) 1, (**c**) 5, and (**d**) 9%] channels. A drain voltage of 0.1 V was applied.

Figure 8 shows transfer characteristics of the TFTs with poly-InO*_x_* and poly-InO*_x_*:H channels. The field effect mobility (μ_FE_) was extracted from the linear region with a drain voltage of 0.1 V. The gate leakage current of all the TFTs at a gate voltage of 20 V was below 0.1 nA (data not shown), which was approximately 5 orders of magnitude lower than the drain current. Thus, gate leakage current had no effect on the extraction of the μ_FE_. The TFT with a poly-InO*_x_* channel did not show switching properties, as shown in Figure 7a. Since the poly-InO*_x_* film exhibited degenerate metallic conduction with *N*_e_ of 2.8 × 10^19^ cm^−3^, a 50 nm thick channel could not be fully depleted by the gate electric field. In contrast, all the TFTs with poly-InO*_x_*:H channels showed clear switching properties. This result indicated that the penetration depth of the gate electric field significantly increases in the nondegenerate poly-InO*_x_*:H channel upon the transition from metal to semiconductor; thus, the 50 nm thick poly-InO*_x_*:H channels could be fully depleted by the gate electric field.

**Figure 9:**
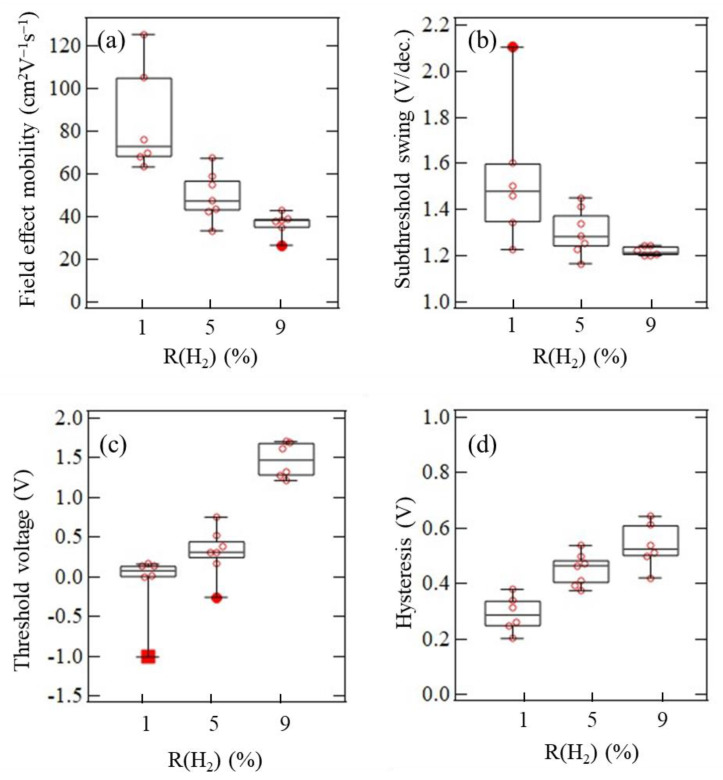
Variations of (**a**) μ_FE_, (**b**) *V*_th_, (**c**) *S.S.*, and (**d**) *V*_H_ evaluated from seven TFTs.

Figure 9 and Table 2 show the variations of the TFT properties evaluated for seven TFTs on the same substrate. From the poly-InO*_x_*:H TFTs with R(H_2_) of 1% and *N*_e_ close to the critical carrier density of the MIT point, the maximum and average μ_FE_ values of 125.7 and 84.7 cm^2^V^−1^s^−1^ were obtained, respectively. Since a hump was often observed in the subthreshold region of high-μ_FE_ TFTs, the variation of a subthreshold swing (*S.S.*) also increased for the TFTs with R(H_2_) of 1%. When R(H_2_) increased, although the average μ_FE_ decreased to 49.7 cm^2^V^−1^s^−1^ and 37.0 cm^2^V^−1^s^−1^ for the TFTs with R(H_2_) values of 5% and 9%, respectively, *S.S.* and its variation became better. In addition, threshold voltage (*V*_th_) shifted positively, but hysteresis (*V*_H_) increased as R(H_2_) increased.

Although further optimization of TFTs and understanding of the role of hydrogen on electrical properties and reliability are still necessary, we successfully demonstrated the formation of high-μ_FE_ TFTs with a nondegenerate poly-InO*_x_*:H channel, formed by SPC. We believe that a nondegenerate polycrystalline InO*_x_*:H channel has great potential for boosting the μ_FE_ of oxide TFTs.

## 4. Conclusions

In this paper, nondegenerate poly-InO*_x_*:H thin films were formed by low-temperature SPC. An a-InO*_x_*:H film deposited by sputtering in Ar, O_2_, and H_2_ gases could be converted to a poly-InO*_x_*:H film by SPC at 250 °C. Hall mobility increased from 49.9 cm^2^V^−1^s^−1^ for an a-InO*_x_* film to 77.2 cm^2^V^−1^s^−1^ for a poly-InO*_x_*:H film. Furthermore, we successfully demonstrated a transition from a metallic poly-InO*_x_* film to a nondegenerate semiconductor poly-InO*_x_*:H film. The carrier density of the poly-InO*_x_*:H film could be reduced to as low as 2.4 × 10^17^ cm^−3^, which was more than two orders of magnitude lower than that of the poly-InO*_x_* film (1.7 × 10^20^ cm^−3^). The TFT with a metallic poly-InO*_x_* channel did not show any switching properties; in contrast, a 50 nm thick nondegenerate InO*_x_*:H channel was fully depleted by a gate electric field. For the InO*_x_*:H TFTs with a channel carrier density close to the critical carrier density of the MIT point, maximum and average μ_FE_ values of 125.7 and 84.7 cm^2^V^−1^s^−1^ were obtained, respectively. We believe that a nondegenerate polycrystalline InO*_x_*:H channel has great potential for boosting the μ_FE_ of oxide TFTs.

## Figures and Tables

**Table 1:** Hall mobility (μ_H_) and carrier density (*N*_e_) of as-deposited and 250 °C annealed InO*_x_* and InO*_x_*:H films.

		As-Deposited		250 °C PDA in N_2_		250 °C PDA in Air	
	R(H_2_) (%)	μ_H_ (cm^2^V^−1^s^−1^)	*N*_e_ (cm^−3^)	μ_H_ (cm^2^V^−1^s^−1^)	*N*_e_ (cm^−3^)	μ_H_ (cm^2^V^−1^s^−1^)	*N*_e_ (cm^−3^)
InO*_x_*	0	44.8	1.7 × 10^20^	46.0	1.1 × 10^20^	26.5	2.8 × 10^19^
InO*_x_*:H	1	49.9	2.1 × 10^20^	77.2	5.1 × 10^19^	27.0	1.4 × 10^18^
	5	32.6	4.6 × 10^20^	75.3	1.2 × 10^20^	13.8	2.4 × 10^17^
	9	17.7	5.8 × 10^20^	74.8	1.0 × 10^20^	20.0	2.7 × 10^17^

**Table 2:** Summary of TFT parameters extracted from seven TFTs on same substrate.

		μ_FE_ (cm^2^V^−1^s^−1^)			*V*_th_ (V)			*S.S.* (V/dec.)			Δ*V*_H_ (V)
	R(H_2_) (%)	Ave.	Max.	Min.	Ave.	Max.	Min.	Ave.	Max.	Min.	Ave
InO*_x_*	0	--	--	--	--	--	--	--	--	--	--
InO*_x_*:H	1	84.7	125.7	63.4	−0.10	0.16	−1.01	1.54	2.10	1.22	0.29
	5	49.7	67.4	33.4	0.31	0.75	−0.26	1.30	1.45	1.16	0.45
	9	36.6	42.9	26.6	1.47	1.70	1.21	1.22	1.25	1.20	0.54

## Data Availability

The data presented in this study are available on request from the corresponding author.
